# Delayed Presentation and Complex Course Secondary to Fish Bone-Induced Esophageal Perforation in an Elderly Patient: A Case Report

**DOI:** 10.7759/cureus.95925

**Published:** 2025-11-01

**Authors:** Deepak Sethumadhavan, Mohamed Zuhail K Peediyakkal, Nabil Mahmood, Awadh A Bintaher, Amit Madhukar Kulkarni, Ashib Thurakkal, Nevin Kannappilly, Saifil Sidhique, Abdulqadir J Nashwan

**Affiliations:** 1 Critical Care Medicine, Hamad Medical Corporation, Doha, QAT; 2 Clinical Imaging, Hamad Medical Corporation, Doha, QAT; 3 Nursing and Midwifery Research, Hamad Medical Corporation, Doha, QAT

**Keywords:** elderly, esophageal perforation, fish bone ingestion, foreign body, mediastinitis, stroke, thoracotomy

## Abstract

Esophageal perforation from foreign body ingestion is rare but potentially life-threatening, especially in elderly patients with comorbidities. Delayed presentation may lead to severe complications, including mediastinitis, sepsis, and shock. A 72-year-old female with a medical history of diabetes mellitus, hypertension, and asthma presented with progressive dysphagia and chest pain, five days after fish bone ingestion. Imaging revealed a linear hyperdense foreign body at T9-T10 and a right mediastinal collection. She underwent thoracotomy and esophageal repair over a T-tube with resection of the affected lung segment. Postoperative complications included a watershed stroke and mediastinal candida infection, requiring tracheostomy, antifungal therapy, VATS drainage, and esophageal stenting. After a prolonged hospital stay, she was discharged in stable condition. This case underscores the importance of early evaluation and multidisciplinary management in elderly patients with suspected esophageal perforation. Delays can result in complex postoperative recovery and severe systemic and infectious complications.

## Introduction

Foreign body ingestion is a frequent emergency department presentation, with fish bones being the most commonly implicated sharp objects due to dietary habits in many regions. While most ingested foreign bodies pass spontaneously, sharp items such as fish bones are more likely to cause impaction and perforation at anatomical narrowings of the esophagus. Esophageal perforation, although rare, is a life-threatening complication with mortality rates ranging between 10% and 40%, depending on the site, severity, and timeliness of management [1].

Delayed presentation of esophageal perforation is particularly dangerous. If diagnosis and treatment are not instituted promptly, especially beyond 24 hours, the risk of mediastinitis, sepsis, and multi-organ dysfunction rises sharply. In elderly patients, the clinical picture may be obscured by atypical symptoms or comorbid conditions, leading to diagnostic delays. Computed tomography (CT) has become the gold standard for diagnosis, offering high sensitivity for detecting foreign bodies, air leaks, and associated collections, thereby guiding timely surgical or endoscopic interventions [2].

The management of esophageal perforation requires an individualized, multidisciplinary approach, ranging from conservative management and endoscopic stenting to surgical repair and drainage. Despite advances in both surgical and endoscopic techniques, delayed diagnosis continues to be associated with high morbidity and prolonged hospitalizations. This case describes a 72-year-old woman with delayed fish bone-induced oesophageal perforation, complicated by mediastinitis, candida infection, stroke, and respiratory failure, highlighting the diagnostic challenges and importance of coordinated multidisciplinary care in elderly patients [3].

## Case presentation

**Figure 1:**
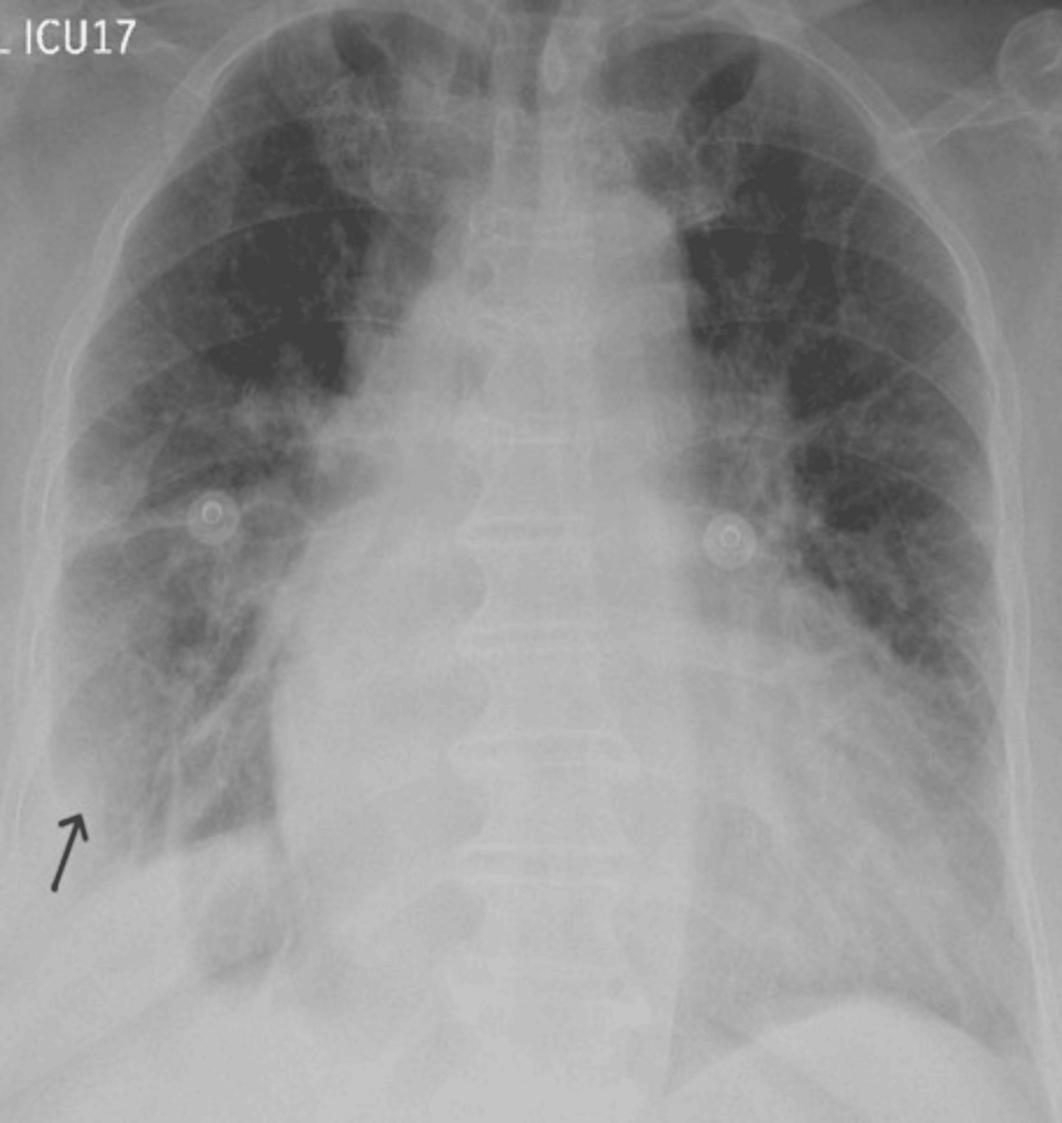
Chest X-ray (anteroposterior view) Chest X-ray (anteroposterior view) showing right lower zone consolidation with patchy airspace opacities and associated blunting of the right costophrenic angle, suggestive of a minimal right pleural effusion (indicated by black arrow).

A 72-year-old female with a history of diabetes mellitus, hypertension, and bronchial asthma presented to the emergency department with right-sided chest pain and shortness of breath. Chest X-ray on admission showed non-homogeneous opacities in the right lower zone (Figure 1). Hence, she was admitted under the internal medicine service with a working diagnosis of community-acquired pneumonia and started on antibiotics.

**Figure 2:**
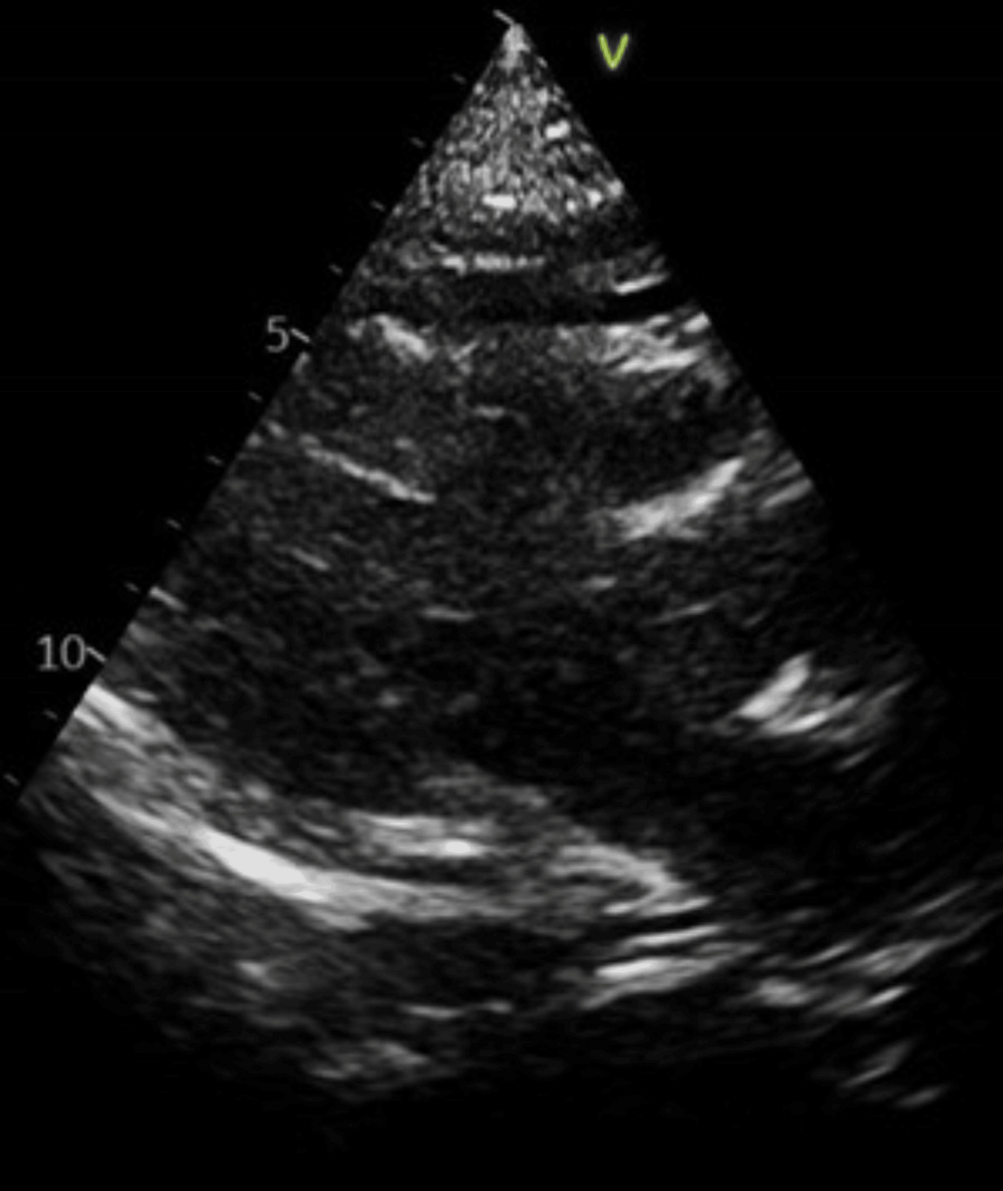
Echocardiogram showing minimal pericardial effusion

Echocardiography performed on admission showed a mild pericardial effusion, and a rare possibility of viral pericarditis was considered as a differential diagnosis (Figure 2).

During hospitalization, she developed new-onset atrial fibrillation with rapid ventricular response, and she was transferred to the intensive care unit (ICU). Her heart rate was controlled with beta blockers and amiodarone, and she was started on therapeutic anticoagulation. In the ICU, on revisiting the history, she admitted to having accidentally ingested a fish bone five days prior to admission. Although she experienced difficulty swallowing and pleuritic chest pain since then, she initially did not seek immediate medical attention.

**Figure 3:**
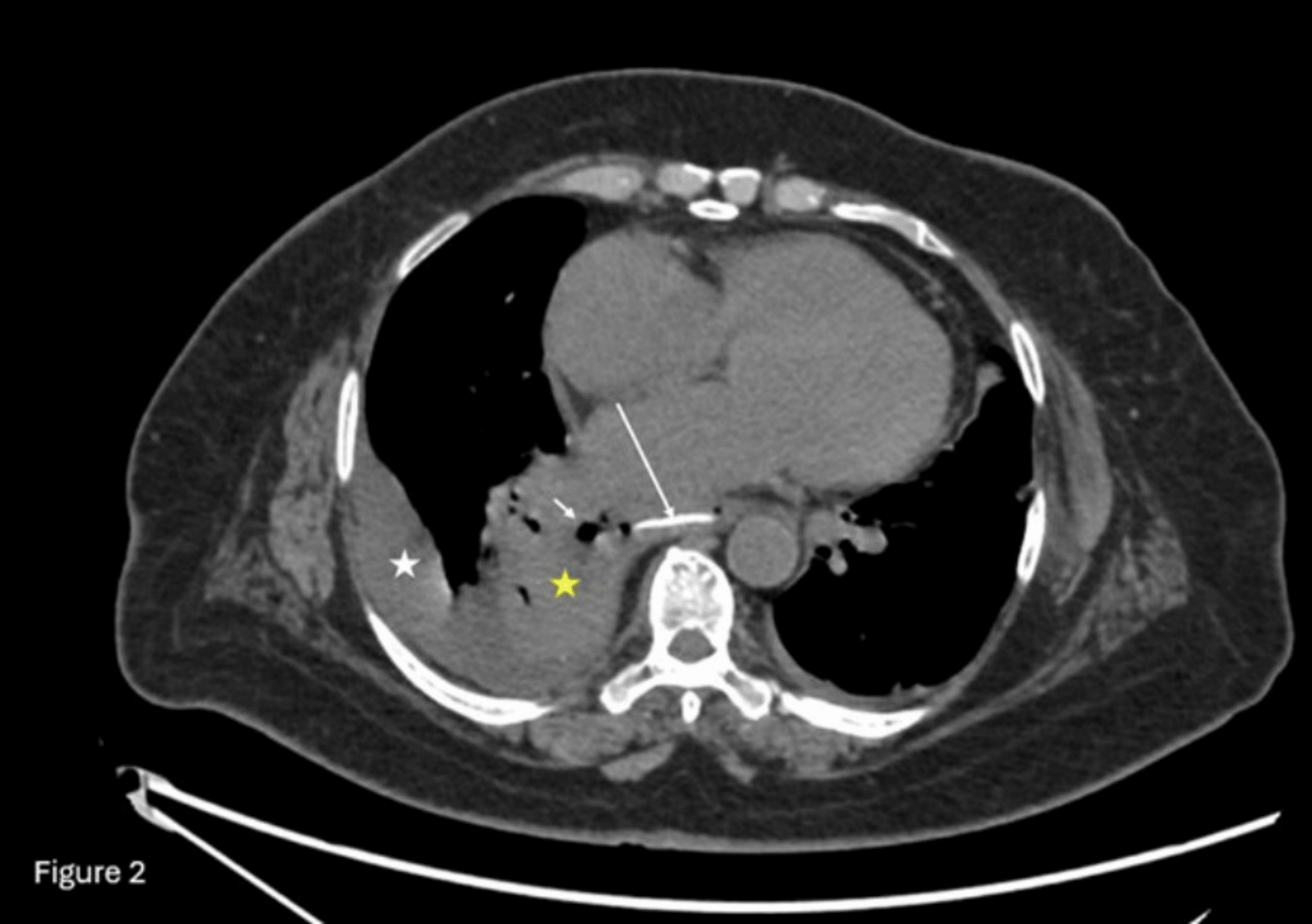
A plain axial CT section of the chest A plain axial CT section of the chest demonstrates a linear hyperdense foreign body consistent with a fish bone in the lower esophagus (long white arrow). Paraesophageal mediastinal air locules (short white arrow) are noted, with an associated right-sided pleural effusion (white asterisk) and underlying consolidation (yellow asterisk).

**Figure 4:**
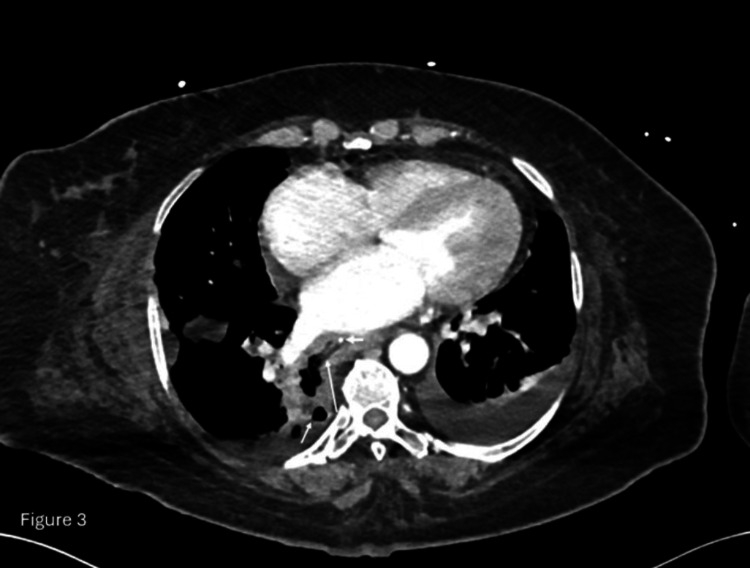
Axial CT section of the chest with intravenous contrast Axial CT section of the chest with intravenous contrast following removal of the fish bone depicts a clear defect in the right lateral wall of the thoracic esophagus (long white arrow) communicating with the right pleural cavity, which contains fluid and air locules (short white arrow). A nasogastric tube is noted within the esophagus (bold white arrow).

A CT scan of the chest was performed for further evaluation, revealing a 23 mm hyperdense linear object in the lower thoracic esophagus (T9-T10) and a right apical mediastinal collection with air bubbles, suggestive of esophageal perforation (Figures 3, 4).

**Figure 5:**
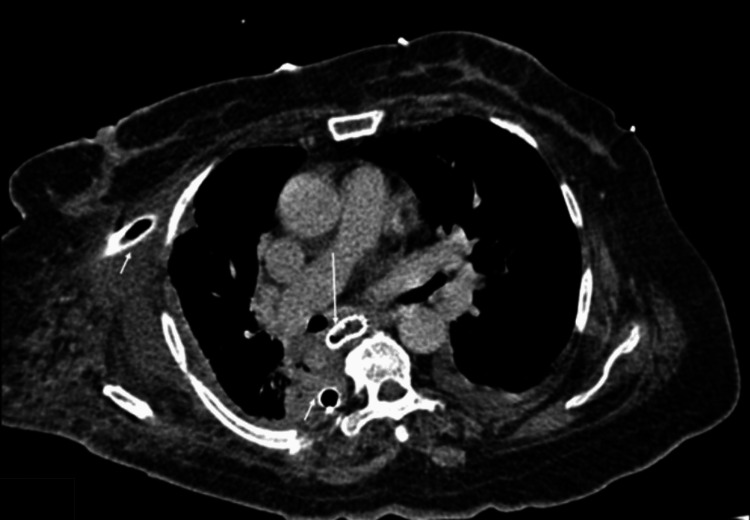
Axial CT section of the chest with intravenous contrast Axial CT section of the chest with intravenous contrast demonstrates an esophageal stent in place (long white arrow). Right-sided chest drains are noted (short white arrows). There is a reduction in the right-sided pleural effusion with resolution of the gas locules.

The next day, she underwent thoracotomy. Intraoperative esophagogastroduodenoscopy revealed an impacted fish bone with purulent discharge and bilateral esophageal perforations. There were lung adhesions, serous effusion, and a localized abscess between the esophagus and right lower lung lobe. Perforations were repaired over a T-tube, and an esophageal stent was placed (Figure 5).

The postoperative period was complicated. The patient developed a left-sided middle cerebral artery (MCA) watershed infarct. She failed multiple extubation attempts and subsequently underwent tracheostomy.

Her postoperative course was further complicated by *Candida mediastinitis* and bacteremia, which were treated with antifungal and broad-spectrum antibiotic therapy (anidulafungin and meropenem).

Table 1 summarizes the microbiological culture results for a patient with recurrent *Candida guilliermondii* isolation from multiple specimen types collected between February 7 and February 15, 2024. Positive cultures were obtained from tissue, catheter tip, tracheal aspirate, wound, and body fluid samples, all confirmed as *Candida guilliermondii* upon microbiological review. Notably, a *Candida auris* screening culture was also performed and later discontinued. The consistent isolation of *Candida guilliermondii* across diverse clinical sites indicates a possible disseminated or persistent infection requiring targeted antifungal management.

**Table 1:** Summary of microbiological culture results showing recurrent Candida guilliermondii isolation from multiple specimen types

Collect date/time (AST)	Order	Growth ind.	Result status	Organism	Status
15/02/2024 21:36:00	*Candida auris *screening culture	—	Auth (Verified)	Candida guilliermondii	Discontinued
15/02/2024 12:46:00	Tissue culture	—	Auth (Verified)	Candida guilliermondii	Completed
15/02/2024 12:46:00	Catheter tip culture	—	—	—	Canceled
15/02/2024 12:40:00	Tissue culture	—	Auth (Verified)	Candida guilliermondii	Discontinued
15/02/2024 12:40:00	Catheter tip culture	Review result	Auth (Verified)	Candida guilliermondii	Discontinued
15/02/2024 12:40:00	Tracheal aspirate culture	Review result	Auth (Verified)	Candida guilliermondii	Completed
08/02/2024 12:40:00	Body fluid culture	Review result	Auth (Verified)	Candida guilliermondii	Completed
08/02/2024 12:40:00	Body fluid culture	Review result	Auth (Verified)	Candida guilliermondii	Discontinued
07/02/2024 12:40:00	Wound culture	Review result	Auth (Verified)	Candida guilliermondii	Completed
07/02/2024 12:40:00	Wound culture	Review result	Auth (Verified)	Candida guilliermondii	Completed
07/02/2024 12:40:00	Fungal culture	Review result	Auth (Verified)	Candida guilliermondii	Completed
07/02/2024 12:40:00	Respiratory lower culture	Review result	Auth (Verified)	Candida guilliermondii	Completed

Stent removal was performed via gastroscopy after a few weeks. The patient improved gradually and was successfully weaned off the ventilator. The tracheostomy was decannulated, and she was discharged one month later in stable condition.

## Discussion

Esophageal perforation from ingested fish bones remains an uncommon but serious clinical problem that disproportionately affects older adults and those with comorbidities. Recent systematic reviews reinforce that delayed presentation (>24 hours) is strongly associated with worse outcomes, increased rates of sepsis, prolonged ICU stay, and higher mortality, emphasizing the time-sensitive nature of diagnosis and source control in these patients. Our patient’s five-day delay before disclosure of ingestion and eventual diagnosis mirrors findings that delayed recognition is a principal driver of complication severity [4].

Diagnosis in this setting relies heavily on cross-sectional imaging. CT scanning provides high sensitivity for detecting radiopaque foreign bodies, mediastinal air, and localized collections. It is now widely considered the imaging modality of choice when esophageal perforation is suspected. In the present case, CT accurately localized the linear hyperdense fish bone and delineated the associated mediastinal collection and pleural involvement, findings that directly informed the decision to proceed to thoracotomy and targeted source control. These observations align with contemporary case series and guidelines that advocate early CT imaging to guide timely surgical or endoscopic therapy [5].

Therapeutic strategies for thoracic esophageal perforation continue to evolve from exclusively open surgical approaches toward multimodal care that integrates surgery, endoscopic stenting, and minimally invasive drainage when appropriate. Systematic analyses and recent reviews document that primary surgical repair with adequate mediastinal/pleural drainage remains indicated for delayed presentations with gross contamination or non-viable tissue. At the same time, endoscopic stenting or endoscopic vacuum therapy (EVT) can be effective adjuncts or alternatives for contained leaks or for patients in whom re-operation carries prohibitive risks [6]. In our patient, thoracotomy with repair over a T-tube plus resection of necrotic lung tissue was necessary for source control; persistent leak thereafter was managed with VATS drainage and an endoscopic stenting approach consistent with the contemporary, staged, multidisciplinary strategies advocated in recent literature [7].

The development of *Candida mediastinitis* in our patient underscores a rare but life-threatening infectious complication after esophageal disruption and prolonged antibiotic exposure. Although bacterial mediastinitis is more commonly reported following esophageal perforation, fungal mediastinitis, particularly due to *Candida *species, has been documented in case reports and small series, often in the setting of extensive contamination, broad-spectrum antibiotic therapy, immunosuppression, or prolonged ICU courses [8]. Early recognition, combined with culture-directed antifungal therapy and surgical or endoscopic source control, is essential for recovery. Our case adds to the body of evidence that *Candida *involvement can complicate an otherwise well-managed esophageal perforation and should be considered when clinical improvement stalls despite antibacterial therapy [9].

Neurological and cardiopulmonary complications further complicated this patient’s recovery, illustrating the multifactorial risks of delayed, extensive thoracic infection in elderly patients. New-onset atrial fibrillation in the setting of sepsis and mediastinal inflammation is common and can necessitate anticoagulation, which in turn complicates surgical and endoscopic management. The watershed cerebral infarct observed in our patient likely reflects a combination of cardioembolic risk from atrial fibrillation, perioperative hemodynamic instability, and the proinflammatory, prothrombotic milieu of severe infection. This intersection of risk factors underscores the need for clinicians to anticipate and manage such complications in similarly complex cases vigilantly. While the literature on perioperative stroke specific to esophageal perforation is limited, broader critical-care and cardiac surgery series document heightened perioperative cerebrovascular risk in elderly, multimorbid patients with sepsis and new arrhythmias [10].

Recent literature supports combining these modalities to maximize leak closure and minimize repeated thoracotomies. Moreover, from a clinical practice perspective, this case highlights three actionable lessons. First, maintain a low threshold for CT imaging in elderly patients presenting with atypical chest pain, dysphagia, or respiratory infection symptoms. Early imaging shortens the time to diagnosis and increases the likelihood of less morbid interventions. Second, adopt a multidisciplinary, staged approach for delayed presentations. This includes aggressive source control (surgical or VATS drainage), targeted antimicrobial or antifungal therapy, and endoscopic adjuncts such as stenting or EVT when indicated. Third, anticipate and proactively manage noninfectious systemic complications (arrhythmia, thromboembolism, and respiratory failure) that substantially affect morbidity and mortality in elderly patients with mediastinal sepsis [11].

## Conclusions

This case illustrates how delayed fish bone-induced esophageal perforation in an elderly patient can precipitate a cascade of thoracic infection, rare fungal mediastinitis, cardiopulmonary instability, and neurological morbidity. The favorable eventual outcome in our patient, despite multiple severe complications, reflects timely escalation to definitive surgical care, the appropriate use of minimally invasive drainage and endoscopic techniques, and aggressive, culture-directed antimicrobial/antifungal therapy coordinated across specialties. Reporting such complex courses contributes to the growing literature that informs triage, imaging, and multimodal management decisions for high-risk patients with esophageal perforation.

## References

[1] Biancari F, D'Andrea V, Paone R (2013). Current treatment and outcome of esophageal perforations in adults: systematic review and meta-analysis of 75 studies. World J Surg.

[2] Kim HU (2016). Oroesophageal fish bone foreign body. Clin Endosc.

[3] Sudarshan M, Elharram M, Spicer J, Mulder D, Ferri LE (2016). Management of esophageal perforation in the endoscopic era: is operative repair still relevant?. Surgery.

[4] Athanassiadi K, Gerazounis M, Metaxas E, Kalantzi N (2002 ). Management of esophageal foreign bodies: a retrospective review of 400 cases. Eur J Cardiothorac Surg.

[5] Pan J, Ge Y, Feng T (2025). Outcome of treatment modalities for spontaneous esophageal rupture: a meta-analysis and case series. Int J Surg.

[6] Aiolfi A, Sozzi A, Bonitta G (2023). Short-term outcomes of different esophagojejunal anastomotic techniques during laparoscopic total gastrectomy: a network meta-analysis. Surg Endosc.

[7] Nachira D, Sassorossi C, Petracca-Ciavarella L (2023). Management of esophageal perforations and postoperative leaks. Ann Esophagus.

[8] Maleb A, Nya F, Amahzoune B, Lemnouer A, Elouennass M (2014). Postoperative mediastinitis due to Candida tropicalis: first reported case in Morocco. J Mycol Med.

[9] Shu L, Jiang W, Xiao H (2023). Perioperative acute ischemic stroke in patients with atrial fibrillation. Ann Neurol.

[10] Dasari BV, Neely D, Kennedy A, Spence G, Rice P, Mackle E, Epanomeritakis E (2014). The role of esophageal stents in the management of esophageal anastomotic leaks and benign esophageal perforations. Ann Surg.

[11] Bhatia P, Fortin D, Inculet RI, Malthaner RA (2011). Current concepts in the management of esophageal perforations: a twenty-seven year Canadian experience. Ann Thorac Surg.

